# Understanding the Uptake of Big Data in Health Care: Protocol for a Multinational Mixed-Methods Study

**DOI:** 10.2196/16779

**Published:** 2020-10-22

**Authors:** Rik Wehrens, Vikrant Sihag, Sandra Sülz, Hilco van Elten, Erik van Raaij, Antoinette de Bont, Anne Marie Weggelaar-Jansen

**Affiliations:** 1 Erasmus School of Health Policy & Management Erasmus University Rotterdam Rotterdam Netherlands; 2 Rotterdam School of Management Erasmus University Rotterdam Rotterdam Netherlands; 3 Department of Industrial Engineering & Innovation Sciences Eindhoven University of Technology Eindhoven Netherlands; 4 School of Medical Physics and Engineering University of Technology Eindhoven Eindhoven Netherlands

**Keywords:** big data, performance, business modeling, regulation, implementation, innovation, social sciences, legitimacy, governmental regulation, balance score card, ethics

## Abstract

**Background:**

Despite the high potential of big data, their applications in health care face many organizational, social, financial, and regulatory challenges. The societal dimensions of big data are underrepresented in much medical research. Little is known about integrating big data applications in the corporate routines of hospitals and other care providers. Equally little is understood about embedding big data applications in daily work practices and how they lead to actual improvements for health care actors, such as patients, care professionals, care providers, information technology companies, payers, and the society.

**Objective:**

This planned study aims to provide an integrated analysis of big data applications, focusing on the interrelations among concrete big data experiments, organizational routines, and relevant systemic and societal dimensions. To understand the similarities and differences between interactions in various contexts, the study covers 12 big data pilot projects in eight European countries, each with its own health care system. Workshops will be held with stakeholders to discuss the findings, our recommendations, and the implementation. Dissemination is supported by visual representations developed to share the knowledge gained.

**Methods:**

This study will utilize a mixed-methods approach that combines performance measurements, interviews, document analysis, and cocreation workshops. Analysis will be structured around the following four key dimensions: performance, embedding, legitimation, and value creation. Data and their interrelations across the dimensions will be synthesized per application and per country.

**Results:**

The study was funded in August 2017. Data collection started in April 2018 and will continue until September 2021. The multidisciplinary focus of this study enables us to combine insights from several social sciences (health policy analysis, business administration, innovation studies, organization studies, ethics, and health services research) to advance a holistic understanding of big data value realization. The multinational character enables comparative analysis across the following eight European countries: Austria, France, Germany, Ireland, the Netherlands, Spain, Sweden, and the United Kingdom. Given that national and organizational contexts change over time, it will not be possible to isolate the factors and actors that explain the implementation of big data applications. The visual representations developed for dissemination purposes will help to reduce complexity and clarify the relations between the various dimensions.

**Conclusions:**

This study will develop an integrated approach to big data applications that considers the interrelations among concrete big data experiments, organizational routines, and relevant systemic and societal dimensions.

**International Registered Report Identifier (IRRID):**

DERR1-10.2196/16779

## Introduction

### Background

The potential of big data in health care is well-recognized in the literature [1-4]. Big data are heterogeneous, complex, and derived from many sources, for example, primary and secondary electronic medical records, laboratory data, prescriptions, imaging data, patient monitors, and telemedicine. Big data can be captured by mobile apps, real-time location tracking, and urban registries [1]. Patients, citizens, and other stakeholders can collect big data, sometimes for other purposes than health care. The framework often used to describe big data is “3V,” which refers to the volume of data, variety of sources and types of data, and velocity of the analysis [5-7]. Other authors have added “veracity” (referring to credibility and “error-free” analytics of big data) and “value” (referring to the impact of big data usage on competitive advantage and performance) to the 3V framework [8-10].

In health care, big data have been described as “encompassing high volume, high variety biological, clinical, environmental, and lifestyle information collected from single individuals to large cohorts, in relation to their health and wellness status, at one or several time points” [11]. With big data *analytics*, routinely generated and collected health care data can be reused for quality improvement (eg, quality registries, benchmarking, and guideline development) [1], population management (eg, early detection of diseases and accessibility), or improved decision making (eg, treatment and cost reduction) [4]. Examples of big data analytics in health care are machine learning, deep learning, image analytics, prediction algorithms, and real-time event detection [4].

In medicine, research has focused on the technical dimensions of big data, that is, how algorithms work and what is technically accomplished with data, and the societal dimensions are underrepresented in medical research literature. Including societal dimensions is important as big data use in health care faces many organizational, social, financial, and regulatory challenges [10,11]. Moreover, organizations have to deal with ethical dilemmas and public outcry [12]. Research on big data technologies and applications in health care should be studied not only as a set of techniques for data extraction, analysis, and reuse, but also as a set of ideas and understandings of its use [13]. Thus, big data research should include the societal dimensions [10-13]. While new analytical techniques may hold significant promise, embedding them sustainably in organizational routines requires more than technical feasibility. It also depends upon “sense-making work” in order to enhance professional acceptability [14]. Similarly, while big data technologies might be legally acceptable, public concerns regarding ethical acceptability can create issues [15]. This protocol therefore outlines an integral research approach that sets out to understand the underlying patterns connected to the societal, business, legal, ethical, political, and organizational change issues surrounding big data applications in health care [10,11].

Limited research in health care has tried to provide an integrated analysis of big data pilots in varying organizational and social contexts. Most papers in this field describe an empirical study of a small-scale pilot, sometimes showing the results of big data analysis [16,17]. For instance, information technology (IT) literature (in health care and beyond) mostly describes promising applications that are yet to be developed [18,19]. In addition, most studies have a specific focus. Business administration literature describes the business value of big data [20] or shows how it can enhance organizational performance [21,22]. The literature in philosophy and ethics centers on theoretical discussions that only occasionally draw on rigorously analyzed empirical examples [15,23]. Importantly, studies in the field of health services research focus on legal frameworks and principles, but often neglect how such frameworks and principles become embedded in organizational practices [24,25]. Hence, little is known about how promising big data pilot projects get integrated in the organizational work practices of care providers or how they get embedded in the daily routines of health care professionals and actually improve health care for all the actors concerned (eg, patients, health care professionals, health care organizations, IT companies, payers, and the society) [26].

A recent systematic literature review revealed that in order to advance our understanding of big data value realization, research should move beyond pilot levels and examine how work practices, organizational models, and stakeholder interests interact with big data technology practices [12]. Few studies provide this integrated analysis of the interaction among the development of concrete pilots, the organizations in which they take place, and the health care systems of which they are a part. Wang et al [27], for instance, developed an integrated transformation model that seeks to investigate causal relationships among big data analytics capability, IT-enabled transformation practices, benefit dimensions, and business values. The authors sought to understand how big data analytics capability transforms organizational practices, thereby generating potential benefits. Cohen et al [28] linked the major legal, policy, and ethical issues raised by predictive analytics to the life cycle phases of predictive analytics models. Heitmueller et al [29] explored questions that policy makers should consider when developing public policy for big data usage in health care. Their approach distinguishes the following three broad categories of barriers: normative barriers (including cultural and ethical norms), market failures, and technocratic barriers (related to technological issues and government processes and regulations). Such studies offer important advances toward an integrated understanding of big data technologies and embedding them in organizations and societies.

In this research protocol, we outline our research approach that aims to add to the body of knowledge on embedding big data applications and technologies in organizations and societies. Applying a mixed-methods approach, our protocol describes how we plan to conduct a detailed multidisciplinary analysis of the interactions among concrete big data applications, organizational routines, and relevant systemic and societal dimensions. We will study how 12 big data applications (developed in pilot projects within a European Union–funded consortium of which we are also a part) strive to become embedded in (1) the daily routines of health care professionals and health care organizations; (2) stakeholder networks in health care organizations with varying infrastructures, policies, routines, and opportunities; and (3) the broader societal context. The overall research question is as follows: How do big data applications and technologies become embedded (or fail to become embedded) in the daily practice of health care professionals, in the health care organization, and in the society at large?

### Research Aim and Approach

This protocol describes research that will be conducted within the context of a broad consortium that is experimenting with 12 big data pilot projects covering the following three themes: population health and chronic disease management, oncology, and industrialization of health care services [30]. The research will aim to understand how big data applications become embedded in the daily practice of professionals (or fail to do so), in the health care organization (why or why not), and in the society at large. To study the interrelations between the applications to be developed in the pilots and the organizational and societal contexts in which they are situated, we will use the Nicolini dual “zooming in-zooming out” approach because “practices are always immersed in a thick texture of interconnections” [31]. We will zoom in to allow different aspects to come to the fore and zoom out to facilitate investigation of the interrelations among concrete big data applications, organizational routines, and relevant systemic and societal dimensions.

To position our work, it is important to note that we will not only study single pilots and their individual contexts, but also feed our insights back into the pilots during the course of the projects. While the idea of transferring gained knowledge in oral and written forms to a pilot shares affinities with action research, our approach differs in two aspects. First, action research traditionally includes *multiple* feedback loops, which not only provide feedback on practical problems but also on any incorporated changes to practice that result from this feedback [32,33]. In contrast, our study focuses on developing knowledge and providing feedback to the pilots, but not on the consequent implementation of this feedback. Second, action research usually focuses on enabling transformative change through a simultaneous process of taking action and doing research, often through a participatory process involving practice members as coresearchers [33]. Instead, our study gives targeted feedback to the pilot, without involving coresearchers in the way action research does. Rather than conducting action research, our study adopts the approach of “situated intervention” by Zuiderent-Jerak [34]. This approach argues that intervention and knowledge production are not opposites, but can be productively combined. Situated intervention builds on the idea that the intervention is not just about practice improvement, but is simultaneously a generative mode of knowledge production. Thus, through commuting between the pilot practices, cocreation workshops with the pilot team members, and theory building beyond the individual pilots, we aim to *both* improve practice via actionable insights and produce new knowledge [35].

We will collect data through a range of carefully aligned studies. We investigate from various disciplinary perspectives how the stakeholders in the 12 pilot projects work toward the performance, embedding, legitimation, and value creation of their big data applications (these dimensions are described below). These pilots all concern innovative uses of big data in health care, but have different purposes and deal with various illnesses and treatments in several contexts (eight European countries with different health care systems). To this end, our study combines methods from many of the social sciences, including health policy analysis, business administration, innovation studies, organization studies, ethics, and health services research. To clarify, this means that our research will not use the big data collected in the 12 pilot projects, but will study how these pilot projects work on dimensions of performance, embedding, legitimation, and value creation.

A multidisciplinary approach is advisable for empirical studies of big data value realization focusing on several dimensions of analysis [12,36]. We will synthesize the data and compare countries to explore similarities and differences in how big data applications become embedded (or not) nationally. To capture the interrelations among individual big data applications, organizational routines, and relevant systemic and societal dimensions, we have selected “performance,” “value creation,” “embedding,” and “legitimation” as key theoretical heuristics. For each of these concepts, we have developed a particular research question (Table 1). In line with our aim to combine study practices and provide feedback, we will visualize the results of each question in easily accessible ways.

**Table 1:** Key concepts and visualization.

Heuristic	Methods	Research question
Performance	Cocreation workshops to develop KPIs^a^ and biannual performance measurements	How does the uptake of big data applications affect health care performance in terms of patient satisfaction, process outcomes, patient outcomes, and financial outcomes?
Embedding	Document analysis, semistructured interviews, focus groups, and follow-up interviews	What underlying mechanisms can explain how big data applications do or do not become embedded in organizational routines?
Legitimation	Document analysis and semistructured interviews	What are the major country-specific facilitators and barriers for the legal, moral, and societal legitimacy of big data applications in health care?
Value creation	Cocreation workshops to develop business models	Which stakeholder group captures which kind(s) of value from big data applications; through which activities, partners, and resources is the value generated; and how can big data applications sustainably be financed?

^a^KPIs: key performance indicators.

*Performance* fits the multidimensional character of public sector organizations [37,38]. The notion of multidimensionality is a central tenet of the distinction between financial and nonfinancial performance, as well as between processes and outputs/outcomes [39]. Following this design, we distinguish patient satisfaction, process outcomes, patient outcomes, and financial outcomes in an adjusted version of the balanced scorecard [40]. Patient satisfaction is defined as the perceptions and experiences of patients with health care delivery and the results thereof. Process outcomes refer to the activities undertaken in health care delivery (eg, hospitalization and visits to the specialist). Patient outcomes are defined as the effects of care on patients’ health status (eg, mortality rates). Financial outcomes are the monetary implications for individuals, organizations, and the society. The associated research question is as follows: How does the uptake of big data applications affect health care performance in terms of patient satisfaction, process outcomes, patient outcomes, and financial outcomes?

The concept of *embedding* refers to the dynamic processes that lead the big data applications developed in the pilots to become integrated (or not) in the daily work practices of health care professionals, organizations, and societies. Based on insights from normalization process theory, we focus on the following four dimensions of embedding: sense-making work (actors’ interpretations of what the application can add to work processes), relational work (actors’ efforts in building a community of practice around the application), operational work (the work of actors involved in establishing new task divisions), and appraisal work (formal and informal assessments conducted by actors to assess the value of the application) [14,41]. Through these four dimensions, we aim to develop insights into the underlying mechanisms of embedding. The associated research question is as follows: What underlying mechanisms can explain how big data applications do or do not become embedded in organizational routines?

We conceptualize *legitimacy* as containing legal, societal, and moral dimensions [42]. The legal dimension refers to whether the big data application complies with formal legislation and official regulations. New regulations (or national policies) can be expected to affect (possibly limit) the opportunities available to the pilots (eg, the EU General Data Protection Regulation [GPDR] legislation could have major consequences for pilots working with international technology development partners). The legal dimension is less straightforward than it appears, as regulations are often diverse and might conflict in practice. Most often legislation trails new technological developments, creating a large gray area in which the application of a regulation can be interpreted in various ways [15]. The societal dimension addresses national policies and the social acceptability of big data. Since regulation is shaped by and embedded in cultural and social practices and policies, these constitute another dimension of legitimacy. The moral dimension focuses on the ethical discussions about big data in different countries. Informal societal aspects (eg, societal perspectives on the sharing of health data, ways in which big data are represented in popular media, and the ethical framing of the debate) can be expected to have consequences for the ways big data applications are developed and legitimized. The associated research question is as follows: What are the major country-specific facilitators and barriers for the legal, moral, and societal legitimacy of big data applications in health care?

The concept of *value creation* relates to the impact of big data applications in terms of both benefits (qualitative or quantitative) and costs for stakeholders (ie, anyone who affects or is affected by the application) [43]. As different stakeholders in the health care system have different perspectives on value [44], the dimensions of value focus on the value needs of the various stakeholders. Value dimensions are broader in scope and thus different from performance dimensions. Moreover, each health care system has its own unique stakeholder network. As such, stakeholder evaluation and participation in the development process will have a distinct inward impact on the success of big data applications in each health care system. We will focus on the value needs of the following various stakeholders usually associated with health care systems: patients, health care organizations and providers, IT companies, vendors, insurers, and the society. The business modeling process suggested by Osterwalder et al can be used to capture, understand, and evaluate the value creation process [45]. Therefore, we will use a business model framework adapted for big data applications in health care to better understand the influence of such applications on the development and implementation of various activities within a health care system. The associated research question is as follows: Which stakeholder group captures which kind(s) of value from each big data application; through which activities, partners, and resources is the value generated; and through which models of cost coverage can big data applications be sustainably financed in different health care systems?

## Methods

Our study base is multidisciplinary to incorporate the many dimensions of big data applications and their mutual interrelations [13,30,36]. The design combines qualitative and quantitative research methods stemming from several social sciences, including health policy analysis, business administration, innovation studies, organization studies, ethics, and health services research [14,15,39,44]. Various researchers with backgrounds in these fields are collaborating in a team that is embedded in the bigger European Union–funded consortium.

The *performance* of big data applications [30] will be monitored over time with pilot-specific key performance indicators (KPIs). It is important that the pilot teams develop their own KPIs with researchers, because these teams possess specific knowledge required to capture the changes in their performance. The KPIs should reflect the multidimensionality of performance underlying this study (patient satisfaction, process outcomes, patient outcomes, and financial outcomes) [46]. We will organize workshops for the 12 pilot teams in order to select KPIs and tailor these to the patient cohort, particular big data application, and aim of the pilot. KPIs will be included in the set if the pilot team and our research group consider them relevant, given the context and availability of reliable data. For the set of relevant and feasible KPIs, data will be collected periodically to allow comparison of performance over time. KPIs are based on various data sources used in the pilots (eg, administrative hospital data, electronic medical records, and registries from regional health ministries). For each pilot, there will be a baseline measurement referring to the period before implementation of the big data application. Following the baseline measurement, data will be collected every 6 months during and after the implementation of the big data application, unless this timing is not feasible or meaningful for the type of pilot.

Since prior literature advocates using dashboards to organize KPIs in a health care setting [eg 47], we will bundle KPIs into dashboards, with visualization per performance dimension to provide feedback to the pilot teams. This visualized feedback will allow both researchers and pilot team members to monitor performance within and across pilots. The information on the KPI dashboards will be discussed periodically with pilot teams to understand performance improvements and obtain insights into the facilitating or hindering factors. At the end of the study period, we will perform a comparative analysis across and within pilots to assess whether or by how much performance has improved during and after the implementation of the big data application. Wherever possible and in close collaboration with the pilots, the comparative analysis will relate to a granular unit of analysis (eg, individual patient level).

To study *embedding*, data are needed from various health care systems, because how rules and regulations are set depends on different actors in the various health care systems [48]. Relevant data to study organizational embedding processes include key contextual documents, such as policy documents from national government and intermediary bodies. At the organizational level, we will analyze organizational strategy documents and conduct semistructured interviews to provide insights into how the different actors and factors involved in setting the pilot influence the embedding of the big data applications in organizational routines. Causal loop diagrams can be used to identify underlying feedback mechanisms that facilitate or hinder these embedding processes [48,49]. Causal loop diagrams derive from a tradition of systems thinking in organizational studies [50] and social sciences [51].

We will study the embedding of big data applications in the following three different health care systems: the Dutch regulated market-based system, Sweden’s decentralized system, and Austria’s national health service. We will perform face-to-face semistructured interviews with pilot members, key organizational actors, and expert informants. The pilot team members will help to identify the respondents who best understand their specific big data pilot and context. At the start of each interview, we will obtain documented consent to record the interview. All recorded interviews will be transcribed verbatim. We will also conduct a document analysis. During the course of the study, pilot team members will collect relevant documents (eg, minutes, policy documents, and relevant emails). The interview material and documents will be qualitatively coded (open, thematic, and axial coding) and analyzed in order to select the 10 to 20 most important factors for a causal model [48,49]. Researchers will draw an initial causal model to explain the hurdles that need to be overcome to structurally embed the big data application in organizational routines, taking country-specific contextual elements into consideration. This initial causal model will be member-checked at a workshop with the pilot team members. After 6 months, we will conduct follow-up interviews with the same respondents to gain new insights into the embedding process. If necessary, we will adapt the causal models to incorporate new developments. We will discuss these new developments for member-checking purposes at the periodic consortium meetings.

Building again on our approach to combine research and intervention [34], the discussions triggered by the causal models will serve as a way to transfer our findings back to the pilot team members, allowing them to use the developed insights in the underlying patterns and mechanisms that hinder or facilitate embedding of big data applications. Specifically, pilot teams could use such insights to improve the embedding process of their big data application.

*Legitimacy* includes legal, societal, and moral dimensions. Previous research by Custers et al [52] identified the following six themes: (1) awareness and trust, (2) government policies for personal data protection, (3) applicable laws and regulations, and (4) their implementation, (5) supervision, and (6) enforcement. Rumbold and Pierscionek [53] compared seven European countries to identify regulatory barriers for restrictions on using health data for research and included both formal legislation and informal social/cultural norms and routines as aspects of informal legitimacy.

We will study the following three aspects of legitimacy: legal (legislation and regulations), societal (social and cultural norms), and moral legitimacy (ethical dimensions and informal norms). Besides conducting document analysis and semistructured expert interviews, we will perform desktop research to analyze policy documents, news articles, scientific papers, presentations, and gray literature for each of the eight countries. We will search for policy strategies on big data or related terms (artificial intelligence and digital health), news articles on big data application in health care, and presentations on the topic given by domain experts. The documents will be qualitatively coded (open, thematic, and axial coding) and analyzed in order to increase our understanding of various country-specific elements, such as the organization of the national health system, concrete examples of media discussions or debates about big data, and specific legislation. We will also conduct 20 semistructured interviews in person or over Skype (Microsoft Corp) per country (n=160) with (1) health care professionals and management; (2) ethical/legal experts; (3) technology/IT developers and data scientists; (4) patient representatives and prominent actors in public/societal debate; and (5) policy makers and other experts. To identify relevant experts in each country, we will build on the knowledge and relationships of the consortium partners, who have an expert network in their country. Other respondents will be identified via document analysis and the snowballing method. At the start of each interview, we will obtain documented consent to record the interview. The topic list will be informed by document analysis and core theoretical concepts from the report by Custers et al [52]. The transcripts of the interviews will be qualitatively coded (open, thematic, and axial coding) and analyzed to develop a detailed understanding of the three dimensions of legitimacy for health-related big data in each country [54].

We will share the results of our aggregated analysis with pilot partners using infographics to visualize the core dimensions of legitimacy for each country, producing insights into the national facilitators and barriers for the uptake of big data applications. Building on our approach to combine research and intervention, the infographics can also be used by pilot partners to support their implementation activities.

We will study *value creation* through cocreation workshops with pilot team members. At these workshops, the researchers will guide team members in developing a business model canvas to gain an understanding of how their big data applications can be made financially sustainable beyond the pilot stage [55]. The business model canvas enables identification of key activities, key resources, required partners, investment and operational costs, (economic) outcomes, and main beneficiaries, as well as the added value of big data for each pilot [56]. The business model canvas will be discussed and refined at periodic meetings. We will collect observational data about the process of business model development, as well as the various versions of the business models as design artifacts. We will analyze the designed business model canvases across the pilots to understand how big data applications impact value creation for various stakeholders across countries and diseases. We will compare business model canvases across the pilots to identify a limited number of business model types for sustainable value creation based on big data applications in health care. The aim is to understand the business prospects of big data in the various national health care systems and under which conditions the business model could be sustainable and add value for patients, providers, payers, and the society.

## Results

The study was funded in August 2017. Data collection started in April 2018 and will continue until September 2021. This combined research approach is likely to lead to the following expected results. First, a set of pilot-specific KPIs and corresponding dashboards to monitor progress. Second, three causal loop diagrams that visualize the underlying patterns and mechanisms that hinder or facilitate embedding of big data applications into broader organizational routines. Third, infographics to visualize the core dimensions of legitimacy for each country, producing insights into the national facilitators and barriers for the uptake of big data applications. Fourth, business model canvases per project to provide insights of value creation.

All our anonymized data will be stored in a secure online environment (BlackBerry Workspace) available to researchers and pilot team leaders only. The study has been approved by the ethics board of Erasmus Medical Center (MEC-2018-056) and the ethics review board of Erasmus University (EA18-01). The review board of Erasmus University checked if we are GDPR compliant. Written informed consent for all participants (including respondents of the interviews, and focus group and workshop participants) will be obtained, and member checks for all interviews will be applied.

## Discussion

### Need for an Integrated Sociotechnical Approach

To evaluate whether big data applications can be embedded in health care systems and provide value for patients, providers, payers, and the society, we need an integrated sociotechnical approach that considers not only concrete experiments but also organizational routines, as well as systemic and societal dimensions. Only then will we be able to develop crucial insights into the interdependencies among big data projects, organizations, and systems required to support providers and payers in their investment decisions and policymakers in shaping their policy goals, ethical questions, and regulations for big data.

### Limitations

Our study is focused on the context of the uptake of big data, concentrating on embedding big data applications and technologies in organizations and societies. However, one could question if health care is already in the embedding phase or still in the phase of understanding how and when to use big data applications, especially by small pilots [57].

### Dissemination of Findings

Our work will be disseminated at conferences and workshops, and published in professional (trade) journals, on electronic media, and in a series of research articles in peer-reviewed journals. We will arrange a series of workshops, inviting stakeholders from the various pilot projects and experts to discuss the contents and the implications of our findings. Dissemination will focus on developing graphic visualizations, as these help to reduce complexity and capture the relations between the dimensions. We will use dashboards to visualize performance over time as measured by jointly developed KPIs.

## Abbreviations

GPDR: General Data Protection Regulation
IT: information technology
KPI: key performance indicator
